# Experimental study of newly described avian malaria parasite *Plasmodium* (*Novyella*) *collidatum* n. sp., genetic lineage pFANTAIL01 obtained from South Asian migrant bird

**DOI:** 10.1186/s12936-021-03588-3

**Published:** 2021-02-10

**Authors:** Elena Platonova, Justė Aželytė, Tatjana Iezhova, Mikas Ilgūnas, Andrey Mukhin, Vaidas Palinauskas

**Affiliations:** 1 Biological Station Rybachy of the Zoological Institute, Russian Academy of Sciences, Kaliningrad Region 238535 Rybachy, Russia; 2 Nature Research Centre, Akademijos 2, LT-09412 Vilnius 21, Lithuania

**Keywords:** *Plasmodium*, Avian malaria, pFANTAIL01, Experimental infection, Rosefinch

## Abstract

**Background:**

Avian malaria parasites are microorganisms parasitizing erythrocytes and various tissues of the birds; they are common and distributed worldwide. These parasites are known to infect birds of different taxa and be the cause of the deaths of birds in the wild and in captivity. The species of parasites with the ability to colonize new territories and infect local non-migratory birds are of particular interest. This scenario is likely in temperate zones of Europe, because of climate change and its contribution in spreading vectors of southern origin, which can be involved in the transmission of malaria parasites. In the present study, a tropical *Plasmodium* parasite from a naturally infected long-distance migrant bird was isolated and tested for its ability to develop in common species of mosquitoes and European short-distance migrant birds.

**Methods:**

*Plasmodium* sp. (pFANTAIL01) was isolated on the Curonian spit of the Baltic sea coast from the naturally infected Common rosefinch, *Carpodacus erythrinus* in June 2019. The parasite was described based on the morphological features of its blood stages, the partial mitochondrial cytochrome *b* gene and development after experimental infection of birds and mosquitoes. The parasite was inoculated into Eurasian siskins, *Carduelis spinus*. Parasitaemia, haematocrit and weight of birds were monitored. At the end of the survey, internal organs were collected to study exoerythrocytic stages of this parasite. Experimental infection of mosquitoes *Culex pipiens* form *molestus* and *Culex quinquefasciatus* was applied to study sporogonic development of the parasite.

**Results:**

Based on morphological features, the parasite was described as a new species, *Plasmodium collidatum* n. sp., and attributed to subgenus *Novyella*. It was revealed that the obtained pFANTAIL01 lineage is a generalist parasite infecting a wide range of avian hosts and most likely is transmitted in South and Southeast (SE) Asia and Oceania. In Europe, this strain was recorded only in adult migratory birds wintering in South Asia. This parasite developed high parasitaemia in experimentally infected siskins and caused 25 % mortality. Exoerythrocytic stages of pFANTAIL01 were found in the lungs, liver, spleen and kidney of the deceased birds. Sporogonic development did not occur in *Cx. pipiens* form *molestus* and *Cx. quinquefasciatus* mosquitoes.

**Conclusions:**

*Plasmodium collidatum* is a highly virulent for Eurasian siskin and completes its development in these birds, which can be considered as a potential vertebrate host if the transmission of the infection starts occurring in Europe and temperate zones.

## Background

Every year billions of European breeding birds migrate to their wintering ground [1]. The distances the individuals cover along their routes can reach thousands of kilometres and inevitably these movements involve birds as reservoirs in transporting and potentially spreading other organisms to the new territories [2, 3]. One group of parasites whose spreading can be enforced by migrating birds causes avian malaria. These parasites belong to Plasmodiidae (order: Haemosporida), are distributed worldwide and are diverse [4]. More than 100 years of studies on *Plasmodium* infecting birds show that some species are virulent to their vertebrate hosts and may cause severe disease [4–10]. Extensive molecular screening of juvenile and adult birds as well as migrant and non-migrating species of birds in Europe reveals which avian malarial parasites are transmitted within Europe and which are exotic species present only in birds after their return from wintering quarters [11]. Among the latter are *Plasmodium delichoni* (genetic lineage pCOLL6), *Plasmodium homonucleophilum* (pSW2), *Plasmodium homocircumflexum* (pCOLL4), *Plasmodium ashfordi* (pGRW2), and some others [12–15]. This is only a small fraction of all recorded *Plasmodium* genetic lineages, which are linked to morphologically described species and contains information about their development and virulence for a vertebrate host. In most of the cases, natural vectors are still unknown.

Transmission of the largest number of potentially invasive avian *Plasmodium* lineages found in Europe, occurs in Africa as this is the main wintering ground for the European long-distance migrant birds [4]. Instead of Africa, several bird species migrate to South Asia and SE Asia, these include Common rosefinch (*Ca. erythrinus*), Rosy starling (*Pastor roseus*), Little bunting (*Emberiza pussila*), Read-breasted flycatcher (*Ficedula parva*), Blyths reed warbler (*Acrocephalus dumetorum*) and a few others [16]. According to the MalAvi database [17], there are more than 20 genetic lineages of avian malarial parasites found in breeding European birds which migrate to South Asia and SE Asia, but only few morphologically described lineages of *Plasmodium* have been identified, e.g. *Plasmodium circumflexum*, pTURDUS1 [18, 19], and *Plasmodium relictum*, pSGS1 [20] and pGRW4 [21]. At present, there are no described malarial parasite lineages linked to *Plasmodium* species, which are transmitted only in South Asia or SE Asia and annually brought to Europe with migrating birds. However, these parasites should be of prime interest as they may become the main threat to local bird populations in the near future. According to some calculations, the prevalence of avian malaria will increase by two-threefold due to global warming [22]. Ecological changes and invasive mosquito species may play the main role in the appearance of new interactions between invasive mosquito species and exotic *Plasmodium* parasites causing the transmission of these parasites on local, non-migrating or within Europe migrating birds. For instance, the invasive Asian tiger mosquito (*Aedes albopictus*), originated from SE Asia, is spreading in some parts of Europe already [23, 24]. The presence of *Plasmodium vaughani* (genetic lineage pSYAT05) DNA was recorded in these mosquitoes collected in Italy [24] and this mosquito could also be a potential vector for other avian malaria parasites, especially those originated from SE Asia [25].

The development and virulence in a vertebrate host and insect vector vary between different *Plasmodium* species and, therefore, identification of the parasite species and knowledge about their biology is crucial to better understand the epizootiology and potential spread of avian malaria. In recent years, the description of newly found avian malaria parasites includes both morphological and phylogenetic information obtained from the molecular examination [13, 15, 26, 27]. Some studies go further and provide information about the development patterns in a vertebrate host, erythrocytic and exoerythrocytic stages, the virulence and information about potential vectors [9, 10, 12].

In the present study, a new species of malaria parasite obtained from a naturally infected long-distance migrant Common rosefinch (wintering in South Asia) in Northern Europe was described. Using morphological and molecular methods the detailed description of blood stages and the phylogenetic relationships of this lineage with other previously described avian malaria parasites is provided. The development in the red blood cells (RBCs) and various tissues together with the caused virulence to the vertebrate hosts was studied on common bird species Eurasian siskins *Cr. spinus*, which migrate within Europe. Sporogonic development of the newly described species was studied in blood sucking mosquitoes *Cx. pipiens* form *molestus* and *Cx. quinquefasciatus*. The obtained data can help understanding new parasite-host associations and impact on the host health in regard to parasite spread, brought on by global climate change and newly formed ecological conditions.

## Methods

### Study site and design of the experiment

In June of 2019, one adult male Common rosefinch was caught using mist-nets on the Curonian Spit of the Baltic Sea (55°09′14 N, 20°51′27 E) at the Biological station “Rybachy” of the Zoological Institute of the Russian Academy of Science (Russia). The blood was collected in heparinized microcapillary by puncturing a brachial vein. Two blood smears were prepared and stained as described by Valkiūnas [4]; about 25–30 µL of blood was stored in SET-buffer (0.05 M Tris, 0.15 M NaCl, 0.5 M EDTA, pH 8.0) for a later molecular analysis. Microscopic examination showed a *Plasmodium* (*Novyella*) sp. infection. The strain was multiplied in one Eurasian siskin by subinoculation of infected blood as described below for studying experimental infection in the vertebrate host. Juvenile Eurasian siskins, a common and widespread in Europe passerine bird, were used. All wild siskins used in the present experiment were captured about 40 km away from the biological station. The mist-nets were established in a mixed forest area near the town Zelenogradsk in the vicinity of Zelenogradka river (54°56’41 N; 20°30’57 E). In all, 18 juvenile siskins were captured and randomly allocated to experimental (8 birds) and control (10 birds) groups after microscopically proving the absence of *Plasmodium* parasites in their blood samples. All birds were checked before experimental infection for haemosporidian parasites using microscopic examination (see chapter below). Later, in the laboratory, blood samples obtained from all birds before the experiment and in the end of the experiment were analysed using PCR-based method (as described below). Experimental birds were housed in individual cages (60 × 40 × 40 cm, Joko GmbH, Germany) and kept in a vector-free aviary under controlled laboratory conditions (room temperature 22 ± 1 °C, photoperiod 17:7 of light:dark). Food and water were provided *ad libitum* during the entire period of the experiment.

To infect experimental birds, standard protocol in accordance with Palinauskas et al. [28] was used. Each experimental bird was subinoculated with a mixture (0.10 mL) of infected blood, 3.7 % sodium citrate and 0.9 % saline in proportion 4:1:5 into the pectoral muscles. Intensity of meronts in the two used donor birds were 0.03 % and 0.05 %. Two birds were inoculated with approximately 5 × 10^4^ number of mature meronts, and six birds received about 8.3 × 10^4^ meronts. Birds from the negative control group were inoculated using the same procedure and blood mixture as the experimental group, but with blood obtained from an uninfected siskin. The duration of the experiment was 36 days. To estimate the development of parasites in the blood the exposed birds were examined every 4 days by taking blood from the brachial vein as was described above. Small drop of blood was used to make smears for microscopy, a fraction of blood (20–30 µL) was placed in SET-buffer for the molecular analysis and the rest (about 30 µL) was used to measure haematocrit level. To measure the haematocrit level, blood collected in capillary was centrifuged for 5 min at 7000 r. p. m using a ELMI CM-70 (ELMI Ltd., Latvia) centrifuge. Also, the body mass was measured in both experimental and control siskins.

At the end of the experiment all experimentally infected birds were euthanized. Their internal organs (the brain, heart, lungs, spleen, liver, kidneys, pectoral muscle) were extracted and placed to a 10 % neutral buffered formalin solution for fixation. Fixed and parafilm-embedded tissues were cut in 4 µm sections, stained with haematoxylin-eosin (H&E) and examined microscopically under 1000 × magnification [4, 10] for parasite’s exoerythrocytic stages. Also smears of the bone marrow from bird’s femurs were prepared. Air-dried films were fixed in absolute methanol for 3 min and stained using Romanowski-Giemsa protocol to check the presence of phanerozoites [4].

### Experimental infection of mosquitoes

To study the development of the new malarial parasite in an invertebrate host, two species of potential vectors, *Cx. pipiens* form *molestus* and *Cx. quinquefasciatus* were used.

Experimental colonies of mosquitoes were established at the Biological Station Rybachy in April 2019. The eggs of mosquitoes were obtained from P. B. Šivickis Laboratory of Parasitology, Nature Research Centre, Vilnius, Lithuania. Insects were kept in isolated laboratory under controlled conditions (room temperature 23 ± 1 °C; humidity 75–80%; photoperiod 17:7 light:dark). Mosquitoes were kept in a nylon netted cage (45 × 45 × 45 cm, BugDorm, UK). Food for mosquitoes was provided in the form of cotton wools saturated with 5% saccharose solution [29].

For experimental infection an infected donor bird with approximate, 1% parasitaemia (gametocytaemia around 0.3%) was used. A bird was carefully immobilized and fixed in a paper tube, leaving only its legs exposed for the mosquitoes [29]. This tube was placed into a separate mosquito cage with about 100 uninfected female mosquitoes taken from the main colony. After 1 h engorged mosquitoes were separated into small cages (17.4 × 17.5 × 17.5 cm) and kept there up to 22 days post exposure (dpe). The same procedure was applied for control group mosquitoes, where a non-infected siskin was used to feed the females obtained from the main colony. Experimental mosquitoes were dissected gradually for preparations of different sporogonic stages. For ookinete preparations mosquitoes were dissected 1–3 dpe, for oocysts 8–22 dpe and for sporozoite preparations 12–22 dpe. Before dissection, all insects were euthanized in an entomological aspirator with cottonwool moistened with 96% ethanol. The preparations of all sporogony stages were made according Žiegytė et al. [30].

In total, 26 *Cx. pipiens* form *molestus* and 26 *Cx. quinquefasciatus* mosquitoes were engorged. In the control group, 25 *Cx. pipiens* form *molestus* had uninfected blood meals.

### Microscopic examination of blood smears and species identification

For blood smears screening, examination of sporogonic stages and parasitaemia calculation in experimental individuals an Olympus CH2O light microscope with × 40 and × 100 magnifications was used. Pictures for measurements of the parasite at its different blood stages were prepared by using the Olympus BX61 light microscope equipped with digital camera DP70. Visualization of pictures was performed using the software AnalySIS FIVE (Olympus Soft Imaging Solutions GmbH, Münster, Germany). Blood smears obtained from wild birds were examined about 15–20 min with × 100 magnification. To evaluate the intensity of parasitaemia, numbers of infected erythrocytes per 10,000 red blood cells were counted [28, 31].

### Molecular examination and phylogenetic analysis

Total DNA was extracted from whole blood stored in SET-buffer, using the ammonium-acetate protocol [32]. The standard nested PCR protocol was used to amplify 478 bp fragment of the mitochondrial cytochrome *b* gene (cyt *b*) of *P. collidatum* [33, 34]. To control for a false amplification one positive control (DNA of *P. relictum* pSGS1) and one negative control (nuclease-free water) were used every10 samples. Final PCR-products were checked for the success of amplification by running them on 2 % agarose gel. Obtained fragments were sequenced from both 5′ and 3′ ends using an ABI PRISM TM 3100 capillary sequencing robot (Applied Biosystems, USA). Obtained sequences were aligned in BioEdit software [35] and identified using BLAST-program of GenBank [36] and MalAvi database [17].

Phylogenetic analysis of the pFANTAIL01 with 28 additional sequences of haemosporidian parasites was conducted using the Bayesian method and performed in MrBayes v.3.1 [37]. The General Time Reversible Model with a proportion of invariable sites and variation among sites (GTR + I + G) was selected by the mrModeltest 3.7 program [38] as the best fitting model. In total, 3 million generations were run with a sample frequency of every 100th generation. Twenty five percent of obtained trees representing the burn-in phase were discarded. Remaining trees were used for the determination of the consensus tree. The final phylogenetic tree was visualized using FigTree software 1.4.4. [39]. The sequence divergence between different lineages was calculated by applying the Jukes–Cantor model of substitution implemented in the program MEGA 6.0 [40].

### Statistical analysis

The statistical analysis was performed by using the RStudio interface based on R software [41]. The normality of distribution in the experimental dataset was evaluated by employing the Shapiro-Wilk test. Wilcoxon rank-sum test was used for the analysis of differences in haematocrit and body mass values between experimental and control groups of siskins. P-value above or equal to 0.05 was considered as significant.

## Results

### Description of parasite

*Plasmodium (Novyella) collidatum* n. sp.

#### DNA-sequence

Mitochondrial cyt *b* lineage pFANTAIL01 (478 bp, GenBank accession no. MW175901).

#### Type host

The Common rosefinch *Ca. erythrinus* (Passeriformes, Fringillidae).

#### Additional hosts

According to literature data the lineage pFANTAIL01 (synonym codes AP63, GenBank accession no. AY714196; C028, DQ212193 and ASI-2012, JX418225) have been recorded in18 species of 15 families in 7 orders of naturally infected birds (see Table 1). Eurasian siskin was susceptible to experimental infection and could be a competent host.

**Table 1:** Host range and distribution of *Plasmodium collidatum* n. sp. (lineage pFANTAIL01) based on molecular examination (places in Results/Description of parasite chapter)

Order and family of the avian host	Species of the avian host	Locality	Reference
*Anseriformes*			
Anatidae	*Dendrocygna javanica*	Unknown	[42]
*Bucerotiformes*			
Bucerotidae	*Penelopides panini*	Philippines	[43]
*Charadriiformes*			
Scolopacidae	*Calidris tenuirostris*	Australia	[44]
*Falconiformes*			
Accipitridae	*Milvus* sp.	Spain	[45]
*Passeriformes*			
Acanthizidae	*Sericornis magnirostris*	Australia	[46]
Fringillidae	*Carpodacus erythrinus*	Czech Republic	[18]
Maluridae	*Malurus coronatus*	Australia	[47]
	*Malurus melanocephalus*	Australia	[47]
Pachycephalidae	*Pachycephala simplex*	Australia	[46]
Petroicidae	*Poecilodryas albispecularis*	Australia	[46]
Rhipiduridae	*Rhipidura rufifrons*	Australia	[42]
Sturnidae	*Acridotheres tristis*	Singapore, Australia	[48, 49]
	*Pastor roseus*	Bulgaria	[50]
Turdidae	*Turdus merula*	India	[51]
Zosteropidae	*Zosterops lateralis*	Australia	[49, 52]
*Psittaciformes*			
Cacatuidae	*Calyptorhynchus funereus*	Australia	[53]
	*Calyptorhynchus lathami*	Australia	[53]
*Strigiformes*			
Strigidae	*Glaucidium cuculoides*	Thailand	[54]

#### Type locality

The Curonian Spit of the Baltic Sea (55°09′14 N, 20°51′27 E).

#### Prevalence

71 Common rosefinches were collected and examined for haemosporidian infection on Curonian spit in 2010–2019 years. About 7% of all rosefinches were infected with different species of blood parasites. *P*. *collidatum* was reported in one bird individual in 2019. According to MalAvi database, this lineage is rare in Europe.

#### Site of infection

**Fig. 1:**
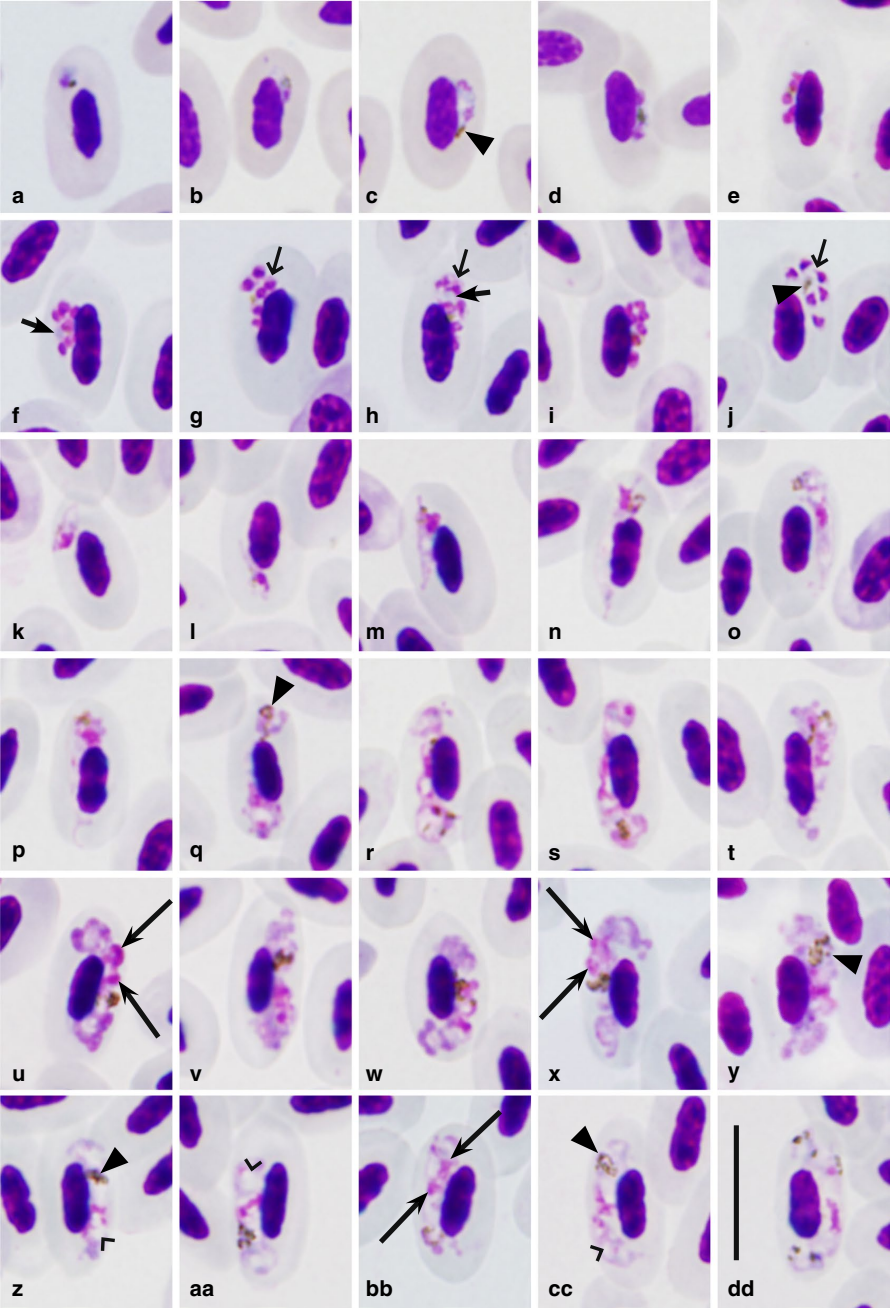
*Plasmodium collidatum* n. sp. (lineage pFANTAIL01) from the blood of the Common rosefinch *Carpodacus erythrinus*: **a**, **b** trophozoites, **c**–**j**—erythrocytic meronts, **k**–**y**—macrogametocytes, **z**–dd—microgametocytes. Long simple arrows—clumps of chromatin; short simple arrows—cytoplasm in meront; triangle heads—pigment granules; simple wide heads—vacuole-like spaces; short simple wide arrows—cytoplasm in merozoites. Giemsa-stained thin blood films. Scale bar = 10 µm

Erythrocytic meronts and gametocytes developed in mature red blood cells (Fig. 1); no other data.

#### Vectors

Natural vectors are unknown. Mosquitoes of *Cx. pipiens* form *molestus* and *Cx. quinquefasciatus* were not susceptible to *P. collidatum*.

#### Distribution

According to the molecular data, *P. collidatum* pFANTAIL01 has been reported in South Asia, SE Asia, Australia, and neighbouring islands as well as in Europe (Table 1). In South Asia, SE Asia and Australia, *P. collidatum* has been found in numerous species of resident and migratory birds. Among infected European birds in the temperate zone, this lineage was confirmed only in adult birds of those species which are wintering in South Asia (*Ca. erythrinus*, *Pastor roseus*).

#### Type specimens

Hapantotype (accession nos. 49,242–49,244 NS, intensity of parasitaemia is approximately 0.3 %, *Ca. erythrinus*, the Curonian spit, Kaliningrad district, Russia, 36° 44′ N, 119° 29′ W, collected 6 June 2019 by E. Platonova) is deposited in the Nature Research Centre, Vilnius, Lithuania. Parahapantotypes (accession nos. G466222, G466223 [1149b/19C, 1220b/19C]), the intensity of parasitaemia is approximately 10 % and 3.3 % respectively, of experimentally infected *Cr. spinus*, collected 26–30 July 2019 by E. Platonova) are deposited in the Queensland Museum, Queensland, Australia.

#### Additional material

Blood films from experimentally infected *Cr. spinus* (accession nos. 1149/19C, 1219/19c, 1220/19C, 1367/19C) and blood samples fixed in SET-buffer (accession nos. 1149/19C, 1219/19c, 1220/19C, 1367/19C) are deposited in the Nature Research Centre, Vilnius, Lithuania.

#### Etymology

The specific name reflects the morphological feature—markedly indented (lobular-like) appearance of the pellicle, which is typical for advanced gametocytes.

#### Exoerythrocytic meronts

**Fig. 2:**
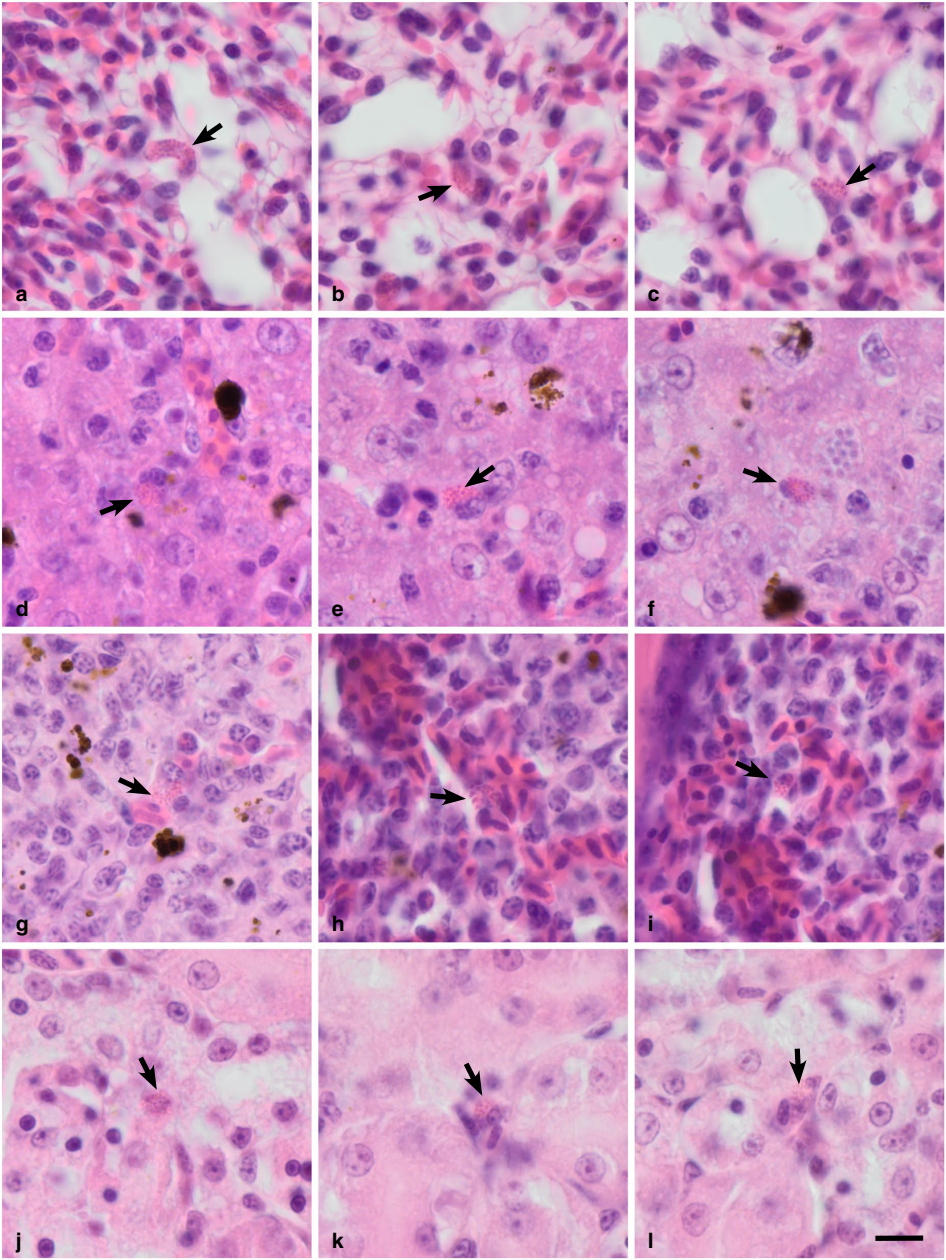
Phanerozoites of *Plasmodium collidatum* n. sp. (lineage pFANTAIL01) from a naturally infected Common rosefinch *Carpodacus erythrinus* in: **a**–**c**—lung, d-f—liver, g–i—spleen, **j**—l—kidney. Arrows indicate the phanerozoites. Haematoxylin and eosin-stained histological sections. Scale bar = 10 µm

Primary exoerythrocytic merogony (cryptozoites and metacryptozoites) was not investigated and remain unknown. Numerous secondary exoerythrocytic meronts (phanerozoites) were found in the lungs (Fig. 2a–c) of the infected individuals and appeared elongated (Fig. 2a), oval (Fig. 2b) or irregularly shaped (Fig. 2c). Phanerozoites in the lungs contained over 30 roundish merozoites. In smaller numbers, phanerozoites were also seen in the liver (Fig. 2d–f), spleen (Fig. 2g–i) and kidneys (Fig. 2j–l) of the infected individuals. In these organs phanerozoites appeared roundish or slightly oval and contained less than 30 merozoites.

**Trophozoites** (Fig. 1a, b) are most often seen in mature erythrocytes, however, rarely can be observed in polychromatophilic red blood cells. Round or oval trophozoites are located at the poles of infected erythrocytes. As trophozoites mature, they attach to the nucleus of the infected erythrocyte and this contact is maintained.

**Erythrocytic meronts** (Fig. 1c–j; Table 2) found only in mature erythrocytes. Growing meronts contain little, but readily visible, blue in colour cytoplasm and small pigment granules (Fig. 1c); in mature meronts cytoplasm is rarely seen. Both, in growing and mature meronts a small bluish roundish and non-refractive globule is often seen (Fig. 1f, h). Meront with developing merozoites touches the erythrocyte nucleus, located laterally or close to the pole of the infected cell and this contact is maintained during the whole time of maturation (Fig. 1c–i). Grown meronts are oval or irregularly shaped and contain 4–8 merozoites (Fig. 1e–i). Pigment granules in mature meronts are small and clumped, however, scattered granules can also be seen in some cells (Fig. 1j). Effect of the gametocyte on the infected erythrocyte is not expressed. Mature merozoites contain prominent irregular-shaped nuclei, and each contains a small portion of readily visible cytoplasm (Fig. 1g, j).

**Table 2:** Morphometry of host cells, mature gametocytes and erythrocytic meronts of *Plasmodium collidatum* n. sp. (lineage pFANTAIL01) (n = 21) (places in Results/Description of parasite chapter)

Feature	Measurements (μm) ^a^
*Uninfected erythrocyte*	
Length	10.4–12.4 (11.5 ± 0.5)
Width	6.1–7.2 (6.5 ± 0.3)
Area	52.8–67.8 (60.5 ± 4.3)
*Uninfected erythrocyte nucleus*	
Length	5.1–6.2 (5.5 ± 0.3)
Width	2.1–2.8 (2.3 ± 0.2)
Area	9.5–14.8 (10.8 ± 1.1)
*Macrogametocyte*	
Infected erythrocyte	
Length	11.2–13.6 (12.4 ± 0.6)
Width	5.3–7.38 (6.1 ± 0.5)
Area	52.4–71.5 (60.4 ± 4.7)
Infected erythrocyte nucleus	
Length	4.6–5.8 (5.2 ± 0.3)
Width	1.6–2.5 (2.1 ± 0.2)
Area	7.0–10.8 (9.0 ± 1.1)
*Gametocyte*	
Length	9.6–13.2 (11.5 ± 1.1)
Width	0.9–2.5 (1.5 ± 0.4)
Area	18.2–28.9 (21.8 ± 2.6)
*Gametocyte nucleus*	
Length	–
Width	–
Area	–
Pigment granules	7.0–14.0 (8.2 ± 1.7)
*Microgametocyte (n = 6)*	
Infected erythrocyte	
Length	10.7–14.1 (12.3 ± 1.3)
Width	6.0–6.6 (6.2 ± 0.2)
Area	53.0–70.5 (61.6 ± 7.3)
Infected erythrocyte nucleus	
Length	4.9–5.8 (5.3 ± 0.4)
Width	2.0–2.6 (2.2 ± 0.2)
Area	9.1–10.6 (9.9 ± 0.6)
*Gametocyte*	
Length	10.5–13.0 (12.2 ± 1.0)
Width	1.8–2.3 (2.0 ± 0.2)
Area	17.5–25.5 (21.6 ± 3.0)
*Gametocyte nucleus*	
Length	1.3–3.8 (2.8 ± 1.0)
Width	0.5–0.7 (0.6 ± 0.1)
Area	0.9–1.2 (1.0 ± 0.2)
Pigment granules	– ^b^
*Meront*	
Length	2.8–5.6 (4.4 ± 0.7)
Width	1.0–2.2 (1.7 ± 0.3)
Area	3.7–8.0 (5.5 ± 1.3)
No. of pigment granules	– ^b^
No. of merozoites	4.0–8.0 (5.8 ± 1.2)

^a^ Minimum and maximum values are provided, followed in parentheses by the arithmetic mean and standard deviation

^b^ Pigment granules are clamped and are difficult to calculate

**Macrogametocytes** (Fig. 1k–y; Table 2) found only in mature erythrocytes. The cytoplasm is markedly heterogeneous and is unevenly stained: more dense stained portions of the cytoplasm are intermediated with pale stained portions, which look like large vacuole-like pale-stained spaces (Fig. 1q–s, w, y), a characteristic feature of this species. Young forms usually found in the poles of infected erythrocytes. In the earliest growing gametocytes, long outgrowths often appear (Fig. 1l). Gametocytes vary in outline from amoeboid in growing to wavy in mature parasites. Gametocytes are located laterally to the nucleus of the infected erythrocyte; maturing and mature gametocytes are strictly nucleophilic, however, growing forms not touching the nucleus can also be seen occasionally (Fig. 1o). A central part of the pellicle of some growing gametocytes does not extend to erythrocyte envelope causing a ‘dip’, which gives dumbbell-shaped form (Fig. 1q, r). Typically, the growing gametocytes are asymmetric in appearance, with one end being broader than the other one (Fig. 1p, s, t, x). Fully-grown gametocytes often do not adhere to the envelope of the erythrocyte and do not fill the poles of erythrocytes (Fig. 1x, y). Markedly wavy lobular-like appearance of the pellicle (on the opposite side to erythrocytes nucleus) of mature gametocytes is an important distinctive feature of this species (Fig. 1u, w). The cytoplasm stains more densely in lobules than in indented areas. The nucleus of the gametocyte is diffused and of unclear outline; it consists of several chromatin clumps, which visually look to be non-connected with each other in Giemsa-stained preparation and can be seen closer to center or anywhere in the gametocyte, a characteristic feature of this species (Fig. 1s–y). Due to the pale cytoplasm staining and diffuse nucleus, macrogametocytes are difficult to distinguish from microgametocytes. Pigment granules are roundish, of small (˂0.5 µm) size, most often grouped in one relatively large distinct spot, but occasionally also were seen scattered in the cytoplasm (Fig. 1s–x). The area of the pigment granule groups is relatively large, and this feature attracts attention during microscopic examination (Fig. 1y). Individual pigment granules do not change size and shape during the development of gametocytes, a rare feature in avian malaria parasites. Effect of the gametocyte on the infected erythrocyte is not expressed.

**Microgametocyte** (Fig. 1z–dd; Table 2) are difficult to distinguish from the macrogametocytes based on the intensity of the cytoplasm staining and appearance of parasite nuclei. The cytoplasm is relatively paler in the microgametocytes, all other characters are as in macrogametocytes. In the blood of type host, microgametocytes were seen extremely rare, and this prevented the complete picture of the morphometry of parasite.

### Taxonomic summary

Morphology of *P. collidatum*—small meronts, a small amount of cytoplasm in meronts and elongated shape of grown gametocytes allows linking this species with the subgenus *Novyella.* Nucleophilic stages developing in the blood of the infected host allows to discriminate this species from other non-nucleophilic species of *Novyella.*This parasite can be readily distinguished from all nucleophilic *Novyella* species due to the following morphological characteristic of its mature gametocytes: (i) the cytoplasm consists of intermediated readily visible large pale-stained and dense-stained areas, providing markedly heterogeneous appearance, (ii) the majority of advanced gametocytes possesses the markedly indented (lobular-like) appearance of pellicle and (iii) macro- and microgametocytes are difficult to distinguish based on their size and morphology of their nuclei. Additionally, the presence of a relatively large loose group on small pigment granules is also helpful during this parasite identification (see our description and [12, 13, 55–57]). The described parasite morphologically is the most similar to *P. delichoni* however, some features, like small roundish granules clearly distinguish this parasite from *P. delichoni*. Other mentioned nucleophilic species also have additional features, which are not found in the described parasite. Growing meronts of *Plasmodium nucleophilum* often displace the nuclei of the infected erythrocytes and its macrogametocytes have a compact nucleus; *Plasmodium paranucleophilum* possesses gametocytes, which push the nucleus in infected erythrocyte laterally; gametocytes of *P. homonucleophilum* are not strictly nucleophilic. None of the above-mentioned features is characteristics of the newly described species. The main morphological features of described parasite blood stages, which were observed in type host (*Ca. erythrinus*) also maintained in Eurasian siskins after experimental infection. Very occasionally meronts with 9 and 10 merozoites appeared in experimentally infected siskins, but not in type host.

### Phylogenetic analysis

**Fig. 3:**
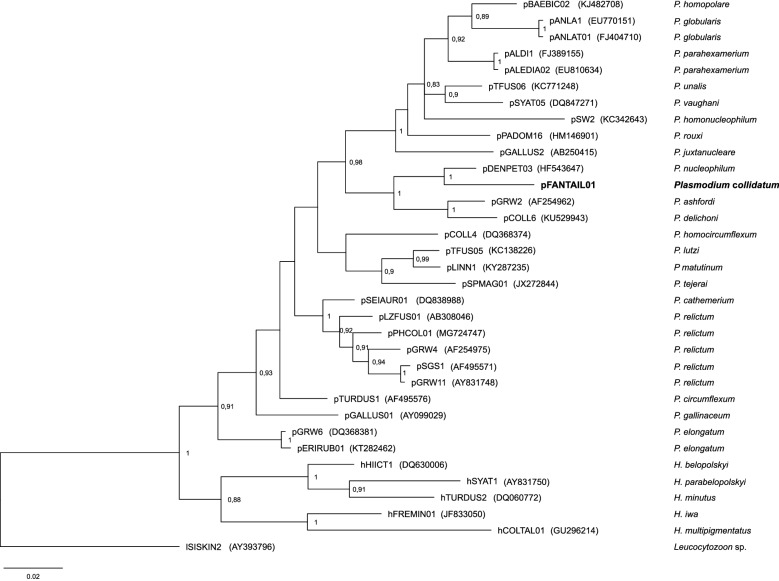
Bayesian phylogenetic tree based on 478 bp mitochondrial cytochrome *b* sequences gene of 28 *Plasmodium* spp. and 5 *Haemoproteus* spp. One genetic lineage sequence of *Leucocytozoon* spp. was used as outgroup. Lineages of parasites and GenBank codes are given in accordance with MalAvi database

According to the phylogenetic analysis, the lineage pFANTAIL01 clusters together with *P. nucleophilum* lineage pDENPET03 (Fig. 3). The genetic distance between these two lineages is 4.31%. Both lineages cluster within a bigger clade with other two *Novyella* parasites e.g. *P. ashfordi* (pGRW2) and *P. delichoni* (pCOLL6). However, the genetic difference between pFANTAIL01 and the latter two cyt *b* lineages is 8.43 and 10.45%, respectively. *Plasmodium collidatum* (pFANTAIL01) morphologically is the most similar to pCOLL6, but distribution and host range of these lineages differ.

### Development and caused virulence in experimentally infected birds

All experimentally exposed siskins were susceptible to *P. collidatum* (pFANTAIL01) and showed the complete development of all blood stages of the parasite. Both PCR and microscopic examination showed that all controls remained uninfected until the end of the experiment.

**Fig. 4:**
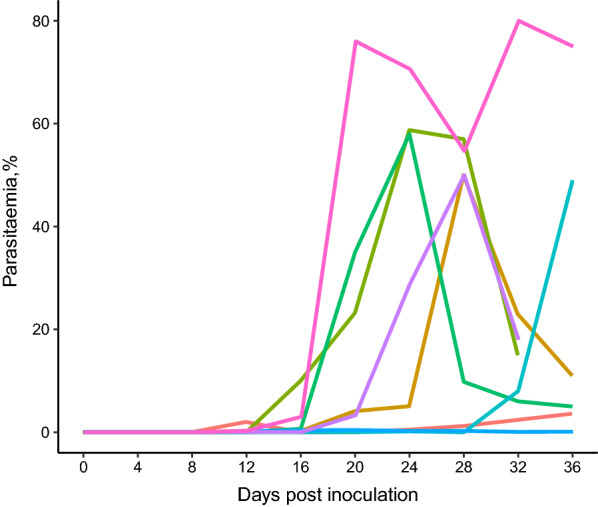
Individual development of parasitaemia of *Plasmodium collidatum* n. sp. (pFANTAIL01) in experimentally infected siskins

**Fig. 5:**
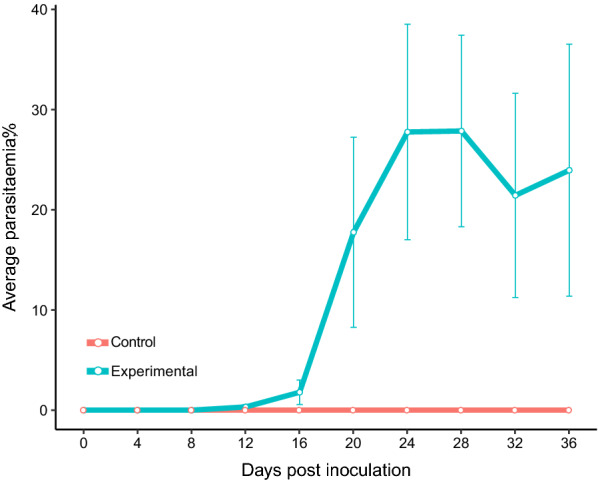
Dynamic of mean parasitaemia of *Plasmodium collidatum* (pFANTAIL01) in experimentally infected siskins. Vertical lines indicate standard error

Prepatent period of infection, when the first infected erythrocytes were detected in the blood smears varied between 12 and 20 days post infection (dpi), (Fig. 4). Dynamics of parasitaemia were highly variable between individuals (Fig. 4); in some reaching up to 70–80%, while in others only < 1%. In half of infected birds after sharp increase of parasitaemia, there was typical bell-shaped form of primary parasitaemia with clearly decreasing slope after the sharp initial increase. High parasitaemia maintained in most of the experimental birds up to 36 dpi, reaching up to 11–75% (Figs. 4 and 5). Two birds died with parasitaemias of 15 and 18% on 32 dpi. All control birds survived until the end of the experiment.

**Fig. 6:**
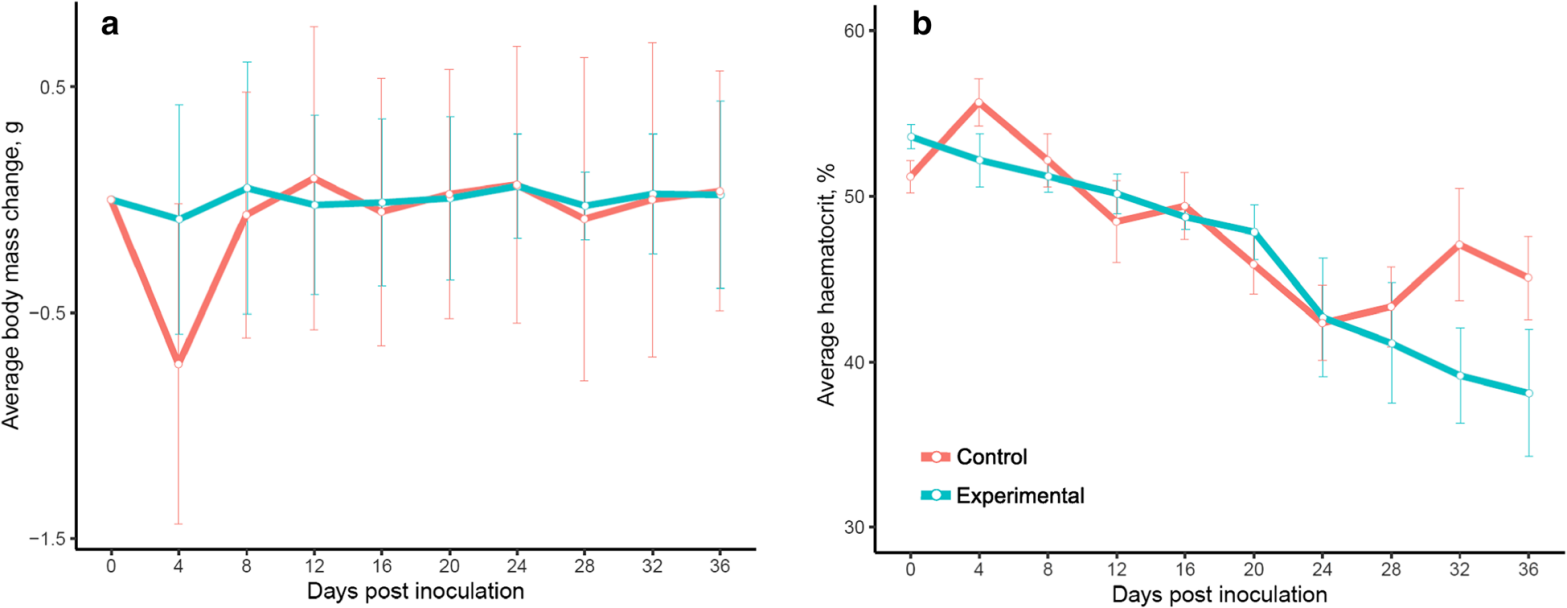
Dynamic of body mass (**a**) and haematocrit (**b**) in experimentally infected and control birds. Vertical lines indicate standard error

The average body mass of the infected birds did not differ significantly from the control group throughout the experiment (W = 4415, p-value = 0.6502, Fig. 6a). The mean haematocrit value was decreasing gradually during the experiment in infected birds, however, there were no significant differences between infected and control siskins (W = 4427, p-value = 0.4631; Fig. 6b). The biggest, but not significant difference between two groups was 32 dpi (W = 53, p-value = 0.08689).

### Development in experimentally infected mosquitoes

After screening *Cx. pipiens* form *molestus* and *Cx. quinquefasciatus* midguts and salivary glands preparations it was determined that ookinetes, oocysts and sporozoites in all exposed to *P. collidatum* (pFANTAIL01) mosquitoes within 22 days after blood feeding were absent. Authors also did not detect any zygotes within 48 h in preparations of mosquito midgut contents.

## Discussion

pFANTAIL01 lineage from the Common rosefinch was identified as *Plasmodium* (*Novyella*) *collidatum* n. sp. The morphological analysis of the erythrocytic stages of the parasite showed that *P. collidatum* has typical features associated with *Novyella* subgenus: elongated gametocytes, small meronts and parasitizing only in mature red blood cells [4]. Based on several unique morphological features of blood stages of pFANTAIL01 lineage it is possible to distinguish it from other *Novyella* parasites and to define this parasite as a separate species (see "Taxonomic summary").

Analysis of accumulated molecular data shows that *P. collidatum* has been recorded predominantly in Oceania and SE Asia (Table 1). It was recorded not only in migrant, but also in resident bird species. The transmission, apparently, takes place in these regions. Several records of pFANTAIL01 were reported in bird species breeding in Europe, Common rosefinch [18], Rosy starling [50] and in one of *Milvus* sp. [45]. All above mentioned passerines were adults after their spring migration returning from South Asia, therefore, it is highly probable that transmission of this parasite does not occur in Europe.

Interestingly, there is one case of *P*. *collidatum* in Spain, from a bird of *Milvus* genus [45]. There might be two species of *Milvus* in Europe, one is *M. milvus* and the second one, *M. migrans* but European populations of both these species do not migrate to South or SE Asia [45]. However, as this is a single record in a raptor bird, it is difficult to make a definite conclusion where and how the bird was infected [58].

According to the MalAvi and GenBank databases, pFANTAIL01 lineage has been reported in wide range of birds of 17 species, 14 families and 7 orders prevailing, herewith, in Passeriformes (Table 1). Apparently, *P. colllidatum* is of low specificity for the vertebrate host and, therefore, can be considered as a generalist species. Although, it is unclear if *P. collidatum* develops gametocytes in all of the mentioned species as the presence of infection in blood was confirmed only by molecular methods and the possibility of abortive parasite development could not be excluded [9, 59].

The damage caused by malaria parasites can be by the tissue stages of exoerythrocytic merogony or by the pathogenic effect due to severe anaemia caused by erythrocytic stages of the parasite [10, 60]. The pathogenic effect of the exoerythrocytic meronts was described in bird species of different families and orders [4, 6, 60] but development and pathogenicity of tissue stages in genera *Novyella* is poorly studied [12, 61]. Phanerozoites were described in *P. nucleophilum toucani* with huge infestation of internal organs and caused mortality in experimentally infected canaries [62]. Phanerozoites were also seen in different internal organs of birds infected by *P. vaughani* [4], *P. paranucleophilum* [57] and *Plasmodium bertii* [4].

The pathogenic effect of the exoerythrocytic stages of *P. collidatum* was reported only in two species of cockatoo, a Yellow-tailed black cockatoo *Calyptorhynchus funereus* and Glossy black cockatoo *Calyptorhynchus lathami* [53]. Both birds were infected in captivity and died despite of the medical treatment. The histological analysis showed a large infestation of schizonts in their liver, spleen, lungs and intestines together with hemorrhage and necrosis of other tissues [53]. Birds died, apparently, due to the extensive damage of their internal organs by the tissue stages of *P. collidatum*. According to the authors, these Australian species live in habitats where the presence of potential vectors is restricted. Both housing cockatoo birds were kept not in their species-specific conditions thus they most likely were exposed to parasite vectors. In the present experiment, numerous phanerozoites were observed in liver, lungs, spleen and kidney tissue of infected siskins. Two of eight birds died at the end of study after decreasing of parasitaemia.

Two mutually non-exclusive factors, the depleted immune system and pathologies caused by phanerozoites could trigger the death of the host. Similar cases have been reported by Ilgūnas et al. [63] in experimentally infected crossbill (*Loxia curvirostra*), siskin and starling (*Sturnus vulgaris*) which were inoculated with a highly virulent parasite *P.* (*Giovannolaia*) *homocircumflexum* (lineage pCOLL4) and mortalities of birds were observed after the peak of parasitaemia.

The negative impact of erythrocytic stages of *Plasmodium* is most noticeable when the parasite damages a big number of blood cells [28, 64–66]. The limited information about the development of *Novyella* parasites is obtained up to now comparing to some *Haemamoeba* or *Giovannolaia* species. It was considered that *Novyella* parasites are mainly of low virulence to birds [67]. However, the studies with infection of tropical origin, *P. ashfordi* (pGRW2) and *P. delichoni* (pCOLL6) showed that these *Novyella* parasites develop high intensities of parasitaemia in experimental birds [12, 15, 68, 69]. According to the present study, siskins are susceptible to *P. collidatum*. The prepatent period varied between individuals but was relatively long in all experimental birds (Fig. 4). This data is in consistent manner with the information about the development of other species of *Novyella* e.g. *P. vaughani* where the prepatent period lasts from 1 to 6 weeks [4, 70], in *P. ashfordi*—2–4 weeks [15], in *P. delichoni*—2–3 weeks [12]. The dynamic of parasitaemia varied among individuals reaching peak values up to 0.42–80% (Fig. 4), but was rather extended in time comparing to other species from the most studied parasites from subgenus *Haemamoeba* which characterized by rapid increase of parasitaemia and rapid decrease to chronic values within 36 dpi [28, 71].

During the study, the impact of *P. collidatum* on body mass and haematocrit level of infected birds was measured (Fig. 6a, b). The negative effect on body mass of the infected siskins was not detected (Fig. 6a). This data agrees with former experimental studies where even severe malaria infection did not affect the body mass of infected individuals, probably, because birds kept in laboratory conditions were receiving food *ad libitum* and were able to compensate the energy loss [10, 28, 72]. Haematocrit level slightly decreased in infected birds on 32 dpi, but the difference between infected and control birds was insignificant (Fig. 6b). This is not a typical case for parasites from other subgenera, because the increase of parasitaemia usually causes the decrease in the number of RBCs [6, 28, 73]. During the infection with *Haemamoeba* parasite *P. relictum* (pSGS1), the quick raise of parasitaemia is immediately followed by a sharp drop of haematocrit value [28]. On the other hand, Palinauskas et al. [9] showed that single infection with low parasitaemia (less than 1%) by *Huffia* subgenus parasite *P. elongatum* (pERIRUB01) dramatically decreased the haematocrit value in experimentally infected siskins. The similar situation was observed in canaries infected with *Novyella* species *P. paranucleophilum*, the bone marrow of infected birds had heavy invasion at low parasitaemia but anemia was clearly manifested by the decrease in haematocrit values [57]. Apparently, a huge infestation of bone marrow by phanerozoites reduces erythropoiesis and, therefore, decrease haematocrit values. At the present study, the exoerythrocytic stages in the bone marrow of the infected siskins were not detected. Probably, the erythropoietic system was compensating the loss of erythrocytes until its depletion on 32 dpi when slight decrease of haematocrit in infected birds but further experimental studies are needed to clarify this point.

The annual migration of birds is an important factor for a possible invasion of new haemosporidian species [4, 74]. However, to complete the life cycle on new territories parasites need a competent vector (Culicidae mosquitoes) and suitable environmental conditions. At the present study, *Cx. pipiens* form *molestus* and *Cx. quinquefasciatus* were used for the experimental investigations. *Culex pipiens* form *molestus* is the common mosquito species distributed around the world and was confirmed as a natural and potential vector for a number of *Plasmodium* species [9, 24, 30, 75, 76]. *Culex quinquefasciatus* is more distributed in subtropical and tropical regions and is known to transmit avian malaria parasites as well [77, 78]. However, mosquitoes of both species experimentally exposed to infection of *P. collidatum* were not susceptible to this parasite. Neither ookinetes, oocysts or sporozoites were detected in any of the exposed insects. Also, zygotes were not seen in blood smears from engorged mosquitoes. Apparently, sporogonic development was aborted on the stage of forming gametes. Further studies are needed to identify a competent vector species for this parasite.

Knowledge about natural vectors of pathogens causing lethal diseases is a cornerstone for the basic understanding of epizootiology of any disease and possible threats in the future. The introduction of competent vector species could lead to the establishment of the tropical *P. collidatum* (pFANTAIL01) in Europe and that could further lead to an outbreak of new malarial infection in local birds which did not co-evolve with the introduced parasite. In the present study, it was experimentally demonstrated that susceptible avian species which could enhance the transmission of tropical pathogen exist in Northern Palearctic.

Despite the fact, that *P. collidatum* did not develop in *Cx. pipiens* form *molestus*, there are other mosquitoes, especially invasive species, which potentially, could serve as a vectors of this parasite. The anthropogenic activity and the global warming are the main factors contributing to the increased numbers of invasive species of mosquitoes and other vectors coming from southern regions [23, 24, 79, 80]. Since recent decades, there are 6 species of mosquitoes and 1 species of biting midges of tropical origin colonizing different parts of Europe. Most of these species are involved in the transmission of various human and animal diseases and could be responsible for the introduction of some of these infections in Europe. For example, the tropical biting midges *Culicoides imicula* introduced the bluetongue virus of ruminants widely throughout Europe [81]. Introduced new mosquito species could serve as competent vectors both for locally already transmitting *Plasmodium* spp. and for exotic blood parasite species carried by long-distance avian migrants. Several field and experimental studies indicated that for instance *Culex sasai* and *Culex pipiens pallens* are competent vectors for some genetic lineages of *Plasmodium* in Asian regions [82, 83]. The introduction of these or other mosquito species from Asia or SE Asia could contribute to transmission of some avian *Plasmodium* parasites, including those which are at present not transmitted in Europe, like *P. collidatum*. However, precise experimental and field studies are needed to determine the possibility of such assumptions.

## Conclusions

In conclusion, a new avian malaria parasite *P. collidatum* n. sp. (pFANTAIL01) was described, which is an incongruous species for European local birds and its transmission takes place in South, SE Asia and Oceania regions. *P. collidatum* completes development in the Eurasian siskin, which is a short-distance migrant within Europe. The parasite develops high intensities of parasitaemia and exoerythrocytic stages in the lungs, liver, spleen and kidneys of the vertebrate host. The negative effect on host health is not expressed in changed body mass and haematocrit values of experimentally infected birds, but this *Plasmodium* species is highly virulent causing the death of infected birds. Although common vector species of avian malaria, *Cx. pipiens* form *molestus* and *Cx. quinquefasciatus* were not susceptible to *P. collidatum*, this parasite should be considered as potential threat to siskins and likely to other non-migrating European birds if suitable vectors and ecological conditions appear.

## Data Availability

All obtained data are available after email inquiry.

## Abbreviations

SE: Southeast Asia
RBC: Red blood cell
Dpi: Days post inoculation
Dpe: Days post exposure
